# Molecular Mechanisms of Dementia 2.0

**DOI:** 10.3390/ijms25137110

**Published:** 2024-06-28

**Authors:** Mariagiovanna Cantone

**Affiliations:** Neurology Unit, Policlinico University Hospital “G. Rodolico-San Marco”, 95123 Catania, Italy; m.cantone@policlinico.unict.it

Dementia and the other neurodegenerative disorders represent a complex pathophysiological process. There is still a pressing need to find suitable biomarkers for the early stages of dementia together with the development and assessment of novel, effective, and customized treatment strategies [1,2], as well as measures to reliably monitor the disease progression and possibly predict the treatment response [3].

Identifying people at a heightened risk for cognitive decline and dementia as early as possible, before symptoms manifest, is the major challenge of modern neurology and molecular neurosciences. In this context, modern molecular biology and neurogenetic techniques may be of particular interest [4,5], although the findings derived from these approaches require caution and careful clinical interpretation [6].

This second edition of the Special Issue of the *International Journal of Molecular Sciences* aims to collect emerging data in patients with memory decline at early stages of cognitive impairment, including mild cognitive impairment (MCI), prodromal dementia, and overt dementia.

Balmorez et al. [7] reported the systematic search of databases for genes involved in Alzheimer’s Disease (AD), aging, and longevity. Including a Reactome analysis that cross-referenced more than 100 bioinformatic databases, the authors identified the genetic interaction networks (referred to as pathways) that are in common between AD and longevity or AD and aging. The overlapped pathways involved gene expression, including ApoE, SOD2, TP53, and TGFB1; protein metabolism and SUMOylation, including E3 ligases and target proteins; ERBB4 signal transduction; the immune system, including IL-3 and IL-13; programmed cell death; and platelet degranulation.

Wang and coworkers investigated the AD-protective APOE allele ε2 functional connectivity (FC) changes in A-PET positive and negative patients using resting state fMRI and graph theory. These authors studied the FC differences in three intrinsic networks (ICNs), including the default mode network (DMN), the salience network (SN), and the central executive network (CEN). The FC of the anterior and posterior DMN was also investigated in different groups. They found that the FC of the anterior DMN (antero-middle cingulate) was lower, and that of the posterior DMN was higher; the SN was not different; and the CEN was higher in the A-PET-positive group than the A-PET-negative group. In terms of the DMN, it is generally known that CNs exhibit increased anterior and decreased posterior functional connectivity in response to Aβ deposition [8].

Um et al. [9] presented an empirical study on sex-related disparities in the resting-state functional connectivity of the locus coeruleus and salience network in 89 cognitively normal patients with evidence of amyloid beta (Aβ) accumulation. 

In the study of Rochin-Hernandez et al. [10], the authors employed microarrays to analyze the transcriptome of nasal epithelial cells of the participants carrying the PSEN1 (A431E) mutation that causes the familial form of AD. They identified the mRNAs and miRNAs with altered levels, determined their target genes at asymptomatic, pre-symptomatic, and symptomatic stages and then further compared them with those from age-matched healthy controls. With the data, they also performed diverse bioinformatic analyses to assess the biological processes and pathways of the differentially expressed genes. The study has the merit of considering the molecular alterations occurring during aging, as it included both younger and older individuals in the analysis. This is of particular importance due to the multi-decade evolution of neurodegenerative diseases and the scarcity of our knowledge on what occurs in the earlier phases of the disease. 

By combining functional analysis with a transcriptome-wide association study, the study of Mamchur et al. [11] evaluated metabolic pathways in different areas of the brain of older adults through a transcriptome-wide association study and functional analysis. The functional analysis revealed a significant association between cognitive impairment and the expression of NADH oxidoreductase in the cerebral cortex. Significant associations were also detected between cognitive impairment and the signaling pathways involved in peroxisome function, apoptosis, and the degradation of lysine and glycan in other brain regions, demonstrating that apoptosis and oxidative stress play important roles in cognitive impairment.

Finally, the study of Almeida et al. [12] broadened the mutation spectrum of GRN and provided an update of the molecular basis of the FTD cohort from the central/north region of Portugal. The identification of the underlying GRN mutations has been essential to provide accurate genetic counseling and clinical management. The frequency of GRN mutations in the cohort was 16%, whereas bvFTD was the most common clinical presentation among the GRN mutation carriers (69%). In addition, among those who underwent neuropsychological evaluation, a neurocognitive profile compatible with a global pattern of moderate-to-severe frontotemporoparietal deficits was found. Moreover, two novel null GRN mutations were identified (c.711delC; p.Thr238Profs*18 and c.1054_1060dupCTCAGCC; p.Leu354Profs*16), with strong evidence of pathogenicity. 
